# Structural mechanism for regulation of DNA binding of BpsR, a *Bordetella* regulator of biofilm formation, by 6-hydroxynicotinic acid

**DOI:** 10.1371/journal.pone.0223387

**Published:** 2019-11-07

**Authors:** William T. Booth, Ryan R. Davis, Rajendar Deora, Thomas Hollis

**Affiliations:** 1 Department of Biochemistry, Center for Structural Biology, Wake Forest School of Medicine, Winston-Salem, NC, United States of America; 2 Department of Microbial Infection and Immunity, and Department of Microbiology, The Ohio State University, Columbus, Ohio, United States of America; University of Queensland, AUSTRALIA

## Abstract

*Bordetella* bacteria are respiratory pathogens of humans, birds, and livestock. *Bordetella pertussis* the causative agent of whopping cough remains a significant health issue. The transcriptional regulator, BpsR, represses a number of *Bordetella* genes relating to virulence, cell adhesion, cell motility, and nicotinic acid metabolism. DNA binding of BpsR is allosterically regulated by interaction with 6-hydroxynicotinic acid (6HNA), the first product in the nicotinic acid degradation pathway. To understand the mechanism of this regulation, we have determined the crystal structures of BpsR and BpsR in complex with 6HNA. The structures reveal that BpsR binding of 6HNA induces a conformational change in the protein to prevent DNA binding. We have also identified homologs of BpsR in other Gram negative bacteria in which the amino acids involved in recognition of 6HNA are conserved, suggesting a similar mechanism for regulating nicotinic acid degradation.

## Introduction

*Bordetella* bacteria are Gram-negative, respiratory pathogens of humans, birds, and animal livestock. Three of the nine currently known species also known as the “classical species” are closely related genetically. *Bordetella bronchiseptica* causes a diverse range of diseases and chronically colonizes four-legged animals, marine mammals and humans [1,2]. *Bordetella pertussis*, the obligate human pathogen responsible for whooping cough, remains a prevalent human health threat in spite of widespread and sustained vaccination coverage [3,4]. *Bordetella parapertussis* can infect both humans and sheep. A significant factor in the persistence of these bacteria is their ability to form biofilms, a sessile lifestyle, in the respiratory tract of infected animals and individuals allowing for efficient spread of the organism between hosts [5–11]. The Bps exopolysaccharide is a critical component of the biofilm matrix and virulence factor of the *Bordetella* species [12–14]. Synthesis of Bps requires the functions of the gene products coded by the *bps*A-*D* locus. Expression of the *bpsA-D* locus is repressed by the transcriptional regulator, BpsR. The BpsR proteins from the three classical *Bordetella* species exhibit >99% amino acid sequence identity [15].

Additionally, BpsR regulates a number of genes relating to cell adhesion, cell motility, cell wall strength, and intra- and extracellular transport [15,16]. Recently, we and others have shown that BpsR controls the growth of *B*. *bronchiseptica* by repressing genes involved in nicotinic acid (NA) degradation [16,17]. Nicotinic acid or nicotinamide is essential for the growth of many pathogenic *Bordetella* species in the laboratory. NA contributes to NAD synthesis in the salvage pathway. It can also serve as a carbon and nitrogen source when degraded through aerobic or anaerobic catabolism. In *Bordetella*, aerobic degradation of NA involves genes in the *nic* cluster, which are conserved in *B*. *bronchiseptica*, *B*. *pertussis*, and *B*. *parapertussis* [16,17] with a similar pathway conserved in other bacteria [18,19]. In the first step of degradation, NA is oxidized to 6-hydroxynicotinic acid (6HNA) by the *nicA* and *nicB* gene products [18,19]. BpsR represses expression of the downstream *nicC* and *nicE* genes [16]. BpsR repression is relieved by binding of 6-hydroxynicotinic acid (6HNA), allowing tight regulation of the pathway to control NA metabolism according to the needs and environment of the bacterium. Thus, deciphering the structural basis of BpsR-HNA interactions is an important step in improving the therapeutic options for disease and infection caused by *Bordetella*.

BpsR is a member of the MarR family of bacterial transcriptional regulators [20]. MarR proteins often repress genes involved in the response to organic compounds, environmental stresses, and virulence factors [21,22]. They are obligate homodimers and many are responsive to ligand binding [23]. While some of the biological aspects of BpsR transcriptional regulation have been reported, its structure and mechanism for allosteric regulation have remained unanswered questions. Here we report the crystal structure of BpsR and the complex of BpsR with 6HNA. These structures reveal a conformational change in protein structure upon BpsR binding of 6HNA that make it incompatible with DNA binding and explain the allosteric regulation.

## Materials and methods

### Cloning, expression, and purification of BpsR

The gene fragment encoding BpsR was inserted into a modified pET19 expression vector (Novagen) which encodes an N-terminal poly-histidine tag, followed by a Rhinovirus 3C protease cleavage site to permit the removal of the affinity tag (PreScission Protease, GE Healthcare). The pET19-*bpsR* vector was transformed into *E*. *coli* strain C41(DE3) cells for expression. One liter of LB-Broth (Luria-Bertani) supplemented with 50 μg/ml of ampicillin was inoculated with 50 ml of an overnight culture of the C41 cells containing the pET19-*bpsR* vector. The cells were grown at 37°C to an OD_600_ = 0.5, and induced with 1 mM isopropyl β-D-thiogalactopyranoside (IPTG) at 16°C for 20 hours. Prior to induction with IPTG, cells were rapidly cooled on ice to 20°C to bring the temperature of the culture close to the induction temperature. Induction of the cells at low temperature was necessary for protein stability during overexpression. Cells were harvested by centrifugation, resuspended in lysis buffer (100 mM Tris pH 7.5, 500 mM NaCl, 5% glycerol, 40mM Imidazole), and lysed using an EmulsiFlex C-5 cell homogenizer (Avestin). Cell debris was removed at 30,000 x g and the supernatant was passed over a Ni-NTA (Qiagen) column equilibrated with lysis buffer. This column was washed with buffer (100 mM Tris pH 7.5, 500 mM NaCl, 5% glycerol, 40 mM imidazole). Bound BpsR was eluted with wash buffer containing imidazole (100 mM Tris pH 7.5, 500 mM NaCl, 5% glycerol, 500 mM imidazole), treated with PreScission Protease according to the manufacturer’s directions, and dialyzed overnight at 4°C against 100 mM MES (pH 6.0), 200 mM NaCl, 5% glycerol, 1 mM dithiothreitol (DTT), and 0.5 mM EDTA. BpsR was further purified using a heparin cation exchange column, and eluted with a 0.1 M– 1.5 M gradient of NaCl. Purity of the peak fractions was verified by SDS-PAGE, and fractions containing pure BpsR were pooled. For crystallization experiments, BpsR was dialyzed against 100 mM Bis-Tris pH 5.5, 100 mM NaCl, 2% glycerol. BpsR was concentrated to 10 mg/mL for crystallization experiments, flash frozen in liquid nitrogen, and stored at -80°C.

### Crystallization of BpsR

Crystallization of BpsR was carried out using the sitting drop vapor diffusion method with equal volumes of protein and crystallization solution in the drop reservoir. Initial crystals were identified in the PEG-ION screen (Hampton Research) and a PEG-infactorial screen [24]. Optimized crystallization conditions contained 18% PEG 2250, 0.2 M potassium formate, and 15% butanediol in the reservoir solution. Crystals were transferred to a drop containing 50% mineral oil, 50% parafin oil (Hampton Research) prior to cryocooling in a liquid nitrogen stream.

Co-crystallization of the 6HNA-BpsR complex was performed with 10 mg/ml BpsR. Crystals were obtained by mixing 2.6 μL of protein solution with 3 μL of the reservoir solution containing 0.1 M HEPES (pH 7.5), 2 mM 6-HNA, 0.2 M MgCl_2_, 18.5% PEG 3350. The crystals were obtained by incubation at 12 ºC.

### Data collection and refinement of the BpsR structure

Diffraction data were collected in house on a Saturn 92 CCD detector or Pilatus3R pixel array detector using Cu Kα radiation from a Micromax007 generator and VariMax optics (Rigaku). Indexing, integration and scaling of the data were performed using HKL3000 program suite [25]. Addition of the 6HNA caused a subtle deterioration in some of the data collection statistics compared to the apo crystal data, however, the CC1/2 of the data (>0.95) suggested the data was of high quality. Phasing of the structure was performed by molecular replacement with the program Phaser, using the structure of a probable MarR transcriptional regulator from *Pseudomonas aeruginosa* as the search model (PDB ID: 2NNN)[26]. The search model was identified using BLAST (blast.ncbi.nlm.nih.gov) as having the highest sequence identity with BpsR (36%) [27]. Manual model building and editing was performed in the program Coot and refinement was carried out using simulated annealing and composite omit procedures using the program Phenix [28,29]. Further model validation and refinement was performed with PDBRedo [30]. Data collection and refinement statistics are listed in Table 1. The atomic coordinates and structure factors have been deposited in the Protein Data Bank with the PDBIDs: 6PCP and 6PCO. The software PyMOL was used for figure preparation [31].

**Table 1:** Data collection and refinement statistics.

	BpsR	6HNA-BpsR
**Wavelength**	1.54	1.54
**Resolution range**	34–2.75 (2.85–2.75)	30–3.2 (3.31–3.2)
**Space group**	P 21 21 21	C 2 2 21
**Unit cell**	71. 9, 90.5, 103.1, 90, 90, 90	77.5, 110.6, 273.3, 90, 90, 90
**Total reflections**	20298 (1866)	117056 (6249)
**Unique reflections**	17964 (1712)	18178 (1564)
**Multiplicity**	1.1 (1.1)	6.4 (4.0)
**Completeness (%)**	98.65 (97.44)	90.78 (79.39)
**Mean I/sigma(I)**	23.40 (4.38)	15.18 (5.71)
**Wilson B-factor**	70.01	45.62
**R-merge**	0.058 (0.549)	0.124 (0.185)
**R-meas**	0.068 (0.642)	0.134 (0.210)
**R-pim**	0.034 (0.330)	0.049 (0.097)
**CC1/2**	0.999 (0.906)	0.989 (0.966)
CC*	0.983 (0.918)	0.997 (0.991)
**Reflections used in refinement**	17793 (1710)	18027 (1537)
**Reflections used for R-free**	911 (75)	1805 (155)
**R-work**	0.2266 (0.3127)	0.2278 (0.2840)
**R-free**	0.2736 (0.3842)	0.2805 (0.3513)
**CC(work)**	0.941 (0.812)	0.936 (0.822)
**CC(free)**	0.938 (0.702)	0.914 (0.757)
**Number of non-hydrogen atoms**	3617	6141
**macromolecules**	3600	6080
**ligands**	6	60
**solvent**	11	1
**Protein residues**	462	834
**RMS(bonds)**	0.002	0.006
**RMS(angles)**	0.61	1.16
**Ramachandran favored (%)**	98.00	98.17
**Ramachandran allowed (%)**	1.33	1.71
**Ramachandran outliers (%)**	0.67	0.12
**Clashscore**	3.00	8.02
**Average B-factor**	72.45	38.71
**macromolecules**	72.56	38.86
**ligands**	47.08	23.09

Statistics for the highest-resolution shell are shown in parentheses.

## Results and discussion

### BpsR structure

**Fig 1:**
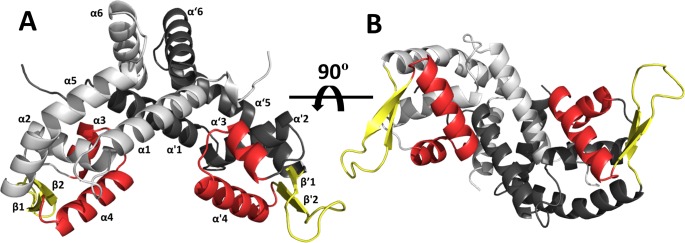
The BpsR structure. **(A)** The monomers of the BpsR dimer are highlighted in light and dark gray, and the secondary structures are labeled (α/α‘ 1–6 and β/β’ 1–2). The conserved winged Helix-turn-Helix (wHtH) domain is highlighted showing the helices in red and the beta hairpin in yellow. **(B)** The BpsR dimer is rotated 90º, showing the DNA binding surface.

BpsR crystallized the in the primitive orthorhombic space group (P2_1_2_1_2_1_) with 2 dimers in the asymmetric unit. The secondary structure of the protein is primarily α-helical composed of 6 α-helices and 2 β-strands per monomer (Fig 1). BpsR contains a winged Helix-turn-Helix (wHtH) domain for DNA binding. In the dimer, the two wHtH motifs are positioned in tandem to one another to create a DNA binding surface (Fig 1). The dimer interface buries about 2355 Å^2^ (PDBePisa (http://www.ebi.ac.uk/pdbe/prot_int/pistart.html [32]), or about 13% of the surface area of each monomer. The overall structure of the dimer is similar to other members of the MarR family of transcriptional regulators [33–37].

**Fig 2:**
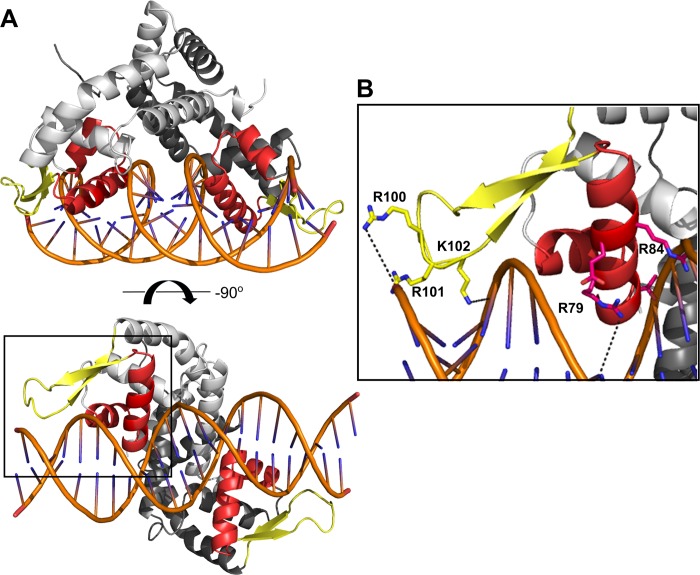
Model of BpsR bound to DNA. The model was created by superimposing the BpsR structure onto the structure of the *Escherichia coli* MarR-DNA complex (PDB ID: 5H3R [38]). The protein structures had an RMSD of 1.37 Å **(A)** This model shows how the recognition helix (red) of the wHtH domain inserts into the major groove of DNA. **(B)** A magnified section of the BpsR-DNA interaction highlights how the residues of the HTH domain interact with the phosphodiester backbone.

Since the precise DNA sequence for BpsR recognition is unclear, it has not been possible to determine a structure in complex with DNA. However, we created a model for BpsR binding to DNA by superimposing the structure of BpsR onto the *Escherichia*. *coli* MarR (ecMarR) structure in complex with a DNA duplex of 21 base pairs (PDB: 5H3R) [38–41] (Fig 2 and S1 Fig). The RMSD between BpsR and ecMarR is 1.38 Å (over 129 Cα atoms) which is indicative of high structural similarity [28]. The model suggests that helix α4, from the HTH domain, inserts into DNA major groove and likely functions in DNA sequence recognition. The β hairpin wing motif extents away from the protein, along the DNA helix, to provide interactions with the phosphodiester backbone and the minor groove (Fig 2B). Although the DNA sequence for BpsR binding is likely different, residues Thr76, Arg79, Gln83, Arg84, Lys86 within helix α4 extend towards the DNA bases within the major groove, providing potential sequence specific interactions. Within the wing region, Arg100, Arg101, and Lys102 (Fig 2B) interact with the DNA phosphate groups in the minor groove also contributing to the stability of the recognition helix interactions within the major groove. Previous data shows that mutation of the corresponding arginine residues in the β-hairpin of other MarR family members, such as OhrR (from *Bacillus subtilis*) and MexR (from *Pseudomonas aeruginosa*) decreased DNA binding 10-fold, which supports their necessity in effective DNA interaction. [33,41,42]

### 6HNA-BpsR bound structure

**Fig 3:**
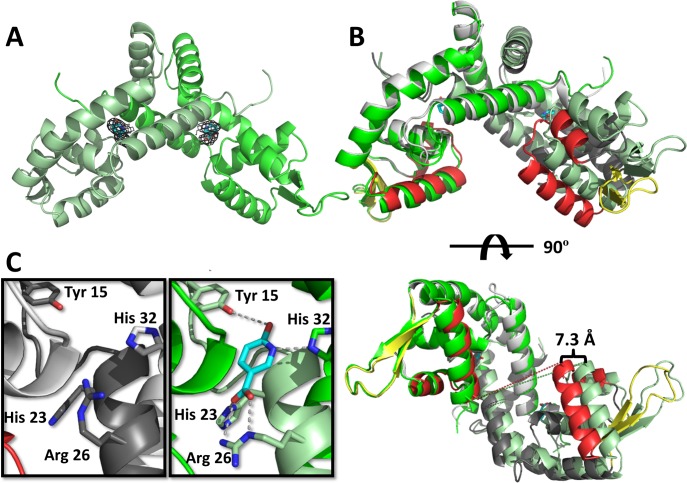
Structure of BpsR bound to 6-HNA (6HNA-BpsR). **(A)** 6HNA-BpsR structure showing the location of the binding pockets within the dimer. The 6HNA 2F_o_-F_c_ map is contoured to 2.0σ. **(B)** The superimposed BpsR (light and dark gray) and 6HNA-BpsR (green) structures reveal a 7.3 Å shift in the location of the recognition helix indicating that the shift prevents DNA binding **(C)** A magnification of the binding pocket of the BpsR (left) and 6HNA-BpsR (right) structures to show the changes that occur with the residues participating in binding of 6HNA. There is a 5 Å rotational shift of Arg26 in order to accommodate 6HNA.

6-hydroxynicotinic acid (6-HNA) binding causes a significant conformational change in the BpsR structure. Recently, we discovered that 6-hydroxynicotinic acid (6HNA) is a negative regulator of BpsR binding to DNA, but the molecular mechanism was not fully understood [16]. To elucidate the structural details of this regulation, we determined the structure of BpsR in complex with 6HNA (Table 1 and Fig 3). 6HNA-BpsR crystallized in the centered orthorhombic space group (C222_1_), with 6 chains in the asymmetric unit. Each dimer bound 2 molecules of 6HNA. The binding pockets are located at the dimer interface between helices α1, α2, α5 of one monomer and α1 of the opposing monomer, and are approximately 8 Å above the recognition helix of the HTH domain (Fig 3A).

A superimposition of the 6-HNA bound and apo BpsR structures reveals a conformational change within the dimer in which the wHtH motif of one monomer pivots away from the other resulting in a 7.3 Å increase in the distance between the α4 DNA recognition helices (Fig 3B). This open conformation with an alteration to the inter-helical distance likely prevents them from inserting into consecutive DNA major grooves thus decreasing binding affinity. A defining characteristic of many MarR family member is allosteric regulation through phenolic like ligands [23], and similarly other MarR family members have a “closed” conformation capable of DNA binding and a “open” form that is unable to bind DNA. Salicylate binding to MarR induces a shift in the protein that widens the dimer into an “open” state that is inactive for DNA binding [36].

**Fig 4:**
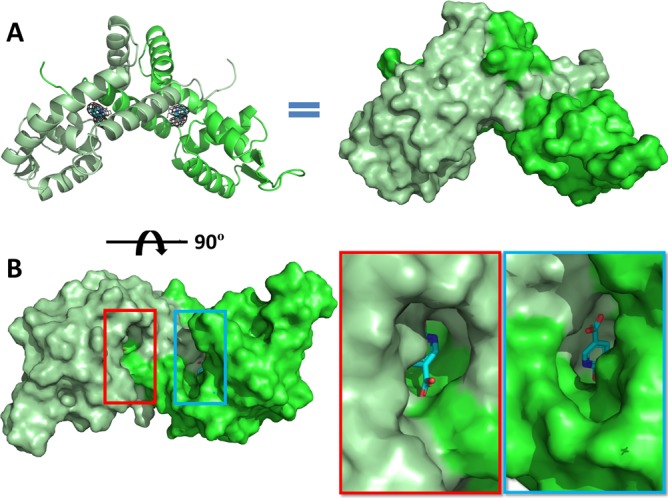
6HNA entry passageway. **(A)** A cartoon illustration of 6HNA-BpsR and the equivalent surface representation. **(B)** The 6HNA-BpsR structure is rotated 90º to show a potential passageway for 6HNA entry in to the allosteric binding site.

6HNA interactions with the protein rearranges the amino acids in the binding site. The bound 6HNA is positioned between four amino acid residues extending from the α1 helix on both monomers. (Fig 3C). Residues Tyr15, His23, and Arg26 from one monomer and His32 from the other monomer each contribute to hydrogen bonding interactions with 6HNA (Fig 3C). A comparison of the conformations of these residues shows that the side chain of Tyr15 shifts approximately 2 Å toward the 6HNA when compared to the unbound structure to H-bond with the 6-hydroxy group of the pyridine ring (Fig 3C). The epsilon nitrogen of His32 contributes to H-bond interactions at the nitrogen on the pyridine ring of 6HNA (Fig 3C). The epsilon and nu nitrogens of Arg26 have H-bonding interactions with the carboxyl group oxygen of 6HNA (Fig 3C). The guanidinium moiety of Arg26 rotates approximately 5 Å away from the binding pocket, using its delta carbon as a hinge, to accommodate the entrance of 6HNA. This residue shows the most significant movement of any sidechain involved in 6HNA interaction (Fig 3C). A protein surface model of the complex shows a potential passageway for 6-HNA entry located directly below the binding pocket (Fig 4).

### 6HNA interactions are conserved

**Fig 5:**
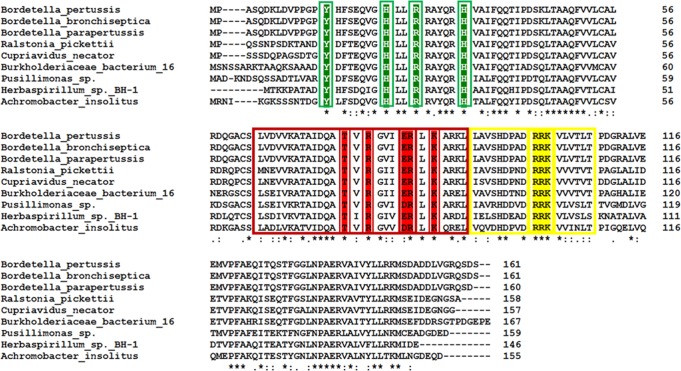
BpsR homologs retain 6HNA binding residues. An amino acid sequence alignment of BpsR homologs from other Gram negative bacteria (Clustal Omega [44]) with an overall sequence identity of about 40%. The residues highlighted in red and yellow boxes are in the HTH and wing domains, respectively. The amino acids highlighted in green are the residues that make up the 6-HNA binding pocket. These residues have 100% identity across the indicated species.

Because the NA catabolism pathway is conserved in some bacteria, we looked to see if the BpsR residues involved in 6HNA interactions are conserved in other bacterial homologs with that also contain the nicotinate dehydrogenase gene (*nicA*) (KEGG Database [43]). We searched for amino acid sequences of proteins similar to BpsR and identified several homologs from at least six other Gram negative bacterial species, many of them also pathogenic (Fig 5 and S1 Fig). The comparative sequence analyses reveal the homologs share about 40% sequence identity across species, with the residues comprising the wHtH domain highly conserved. Interestingly, the residues interacting with 6HNA (Tyr15, His23, Arg26, and His32) are also strictly conserved, suggesting these homologs also may be involved in regulating nicotinic acid metabolism in these organisms. In contrast, the NicR regulator of from *Pseudomonas putida* is also a MarR-like protein and represses *nic* gene expression [18,19]. However, the 6HNA binding residues are not conserved between BpsR and NicR, providing a new model for 6HNA interaction.

Many pathogenic *Bordetella* species have an absolute requirement for nicotinic acid (NA) or nicotinamide for laboratory growth. It serves as a source of NAD synthesis in the salvage pathway and alternatively as a carbon and nitrogen source when degraded. *Bordetella* genes involved in the aerobic degradation of NA are harbored in the *nic* cluster. We have previously shown BpsR binds to the *nic* promoter region, and its DNA binding activity is inhibited by 6HNA, the first metabolite of the NA degradative pathway [16]. We proposed that this regulation by BpsR enables *Bordetella* bacteria to utilize nicotinic acid for their survival depending on environment and metabolic needs.

**Fig 6:**
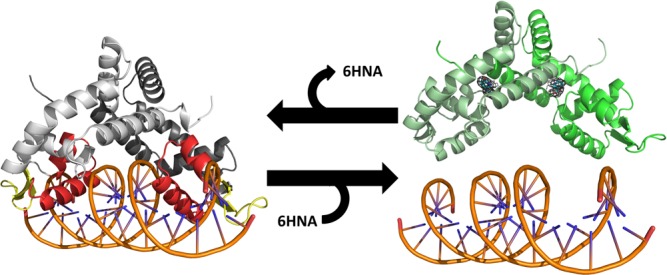
Model for regulation of BpsR regulation. BpsR binding of 6HNA produces a conformational change in the protein that reduces DNA binding affinity.

Here we have determined the structures of BpsR and BpsR in complex with 6HNA in order to understand the molecular mechanism of regulation of DNA binding. The BpsR structure reveal the dimeric architecture of the protein and the overall similarity to the MarR transcriptional regulator. Our structural data along with our previous results lead us to propose a model for regulation of nicotinic acid degradation in Bordetella (Fig 6) in which in the absence of 6HNA, BpsR binds to the *nicC* and *nicE* promoters in the closed form to repress transcription. As nicotinic acid levels increase in the bacteria, expression of the nic genes are induced, resulting in the formation of 6HNA. This molecule then acts as a substrate and inducer of the *nicC* and *nicE* genes by binding to BpsR and inducing a conformational change to an open form that loses DNA binding affinity.

## Supporting information

S1 Fig BpsR amino acid sequence comparisons. A) Sequence alignment of BpsR and E. coli MarR shows 24.5% identity and 52% similarity. B) A phylogenetic tree with real branch lengths showing relationships between BpsR proteins from other Gram negative bacteria (tree produced by Clustal Omega [44]).
(PNG)
