# Supplementary figures and images for: Structural mechanism for regulation of DNA binding of BpsR, a Bordetella regulator of biofilm formation, by 6-hydroxynicotinic acid
Source: PLoS One. 2019 Nov 7;14(11):e0223387. doi: 10.1371/journal.pone.0223387 (PMC6837509; doi:10.1371/journal.pone.0223387)

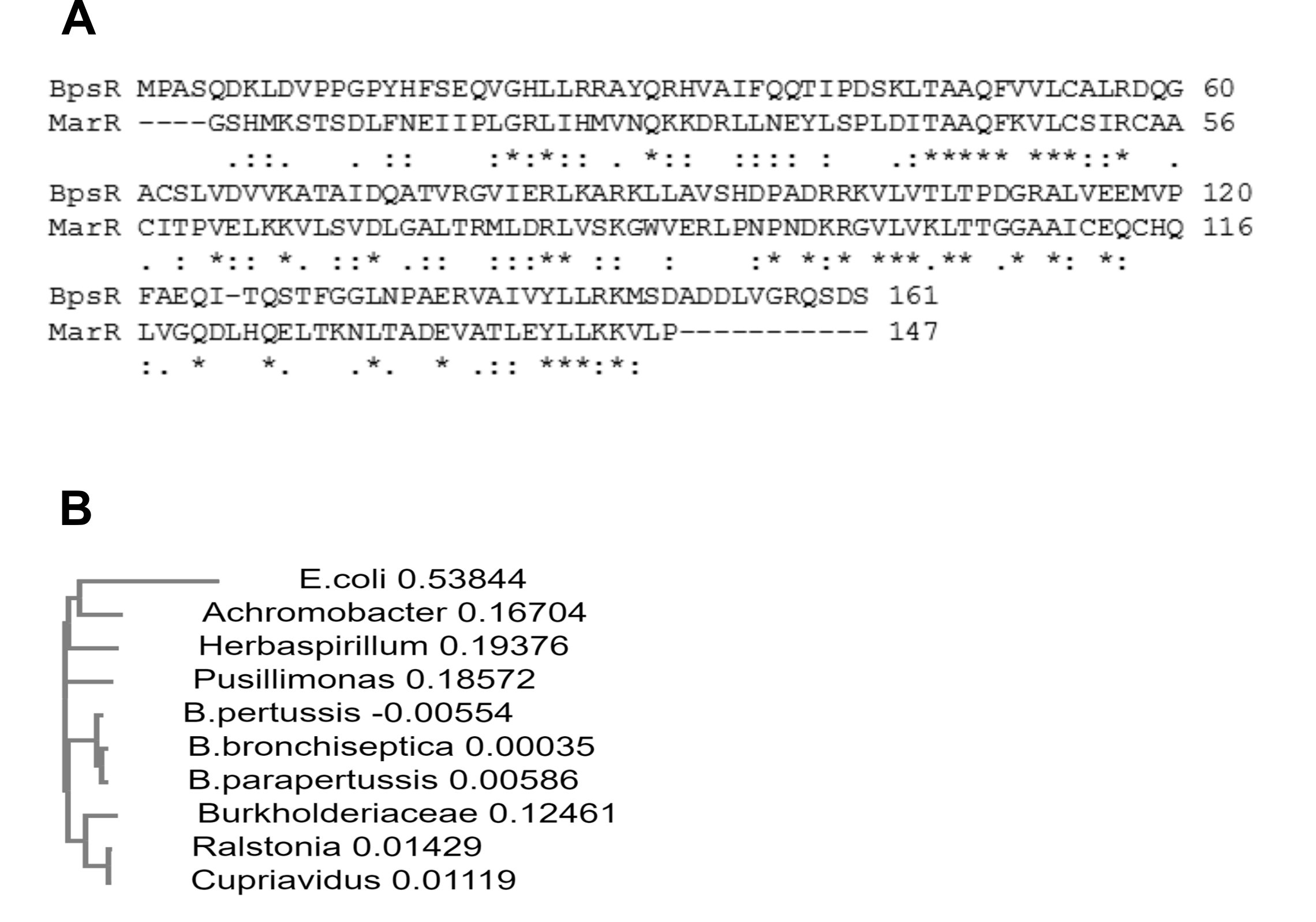

Supplement: S1 Fig — A) Sequence alignment of BpsR and E. coli MarR shows 24.5% identity and 52% similarity. B) A phylogenetic tree with real branch lengths showing relationships between BpsR proteins from other Gram negative bacteria (tree produced by Clustal Omega [44]). (PNG) [file pone.0223387.s001.png]
